# Tea, coffee and risk of glioma

**DOI:** 10.17179/excli2020-2865

**Published:** 2020-09-14

**Authors:** Tomoyuki Kawada

**Affiliations:** 1 Department of Hygiene and Public Health, Nippon Medical School, 1-1-5 Sendagi, Bunkyo-Ku, Tokyo 113-8602, Japan

## ⁯

***Dear Editor,***

Cote et al. (2020[1]) conducted a prospective study to investigate the effect of tea and coffee consumption on glioma risk. No significant associations between tea as well as coffee intake and glioma risk were reported. Here, I will discuss the associations.

Creed et al. (2020[2]) also conducted a prospective study to investigate the effect of coffee and tea consumption on glioma risk. The adjusted hazard ratio (95 % confidence interval [CI]) of four or more cups of tea/day for glioma incidence was 0.69 (0.51-0.94). In contrast, there was no significant association between coffee consumption and subsequent glioma incidence observed. Additional prospective studies are needed to verify the preventive effect of tea consumption on glioma.

Song et al. (2019[5]) conducted a meta-analysis of prospective studies in which the pooled relative risk (RR; 95 % CI) of coffee consumption for brain cancer risk was 0.785 (0.580-0.984). The pooled RR was 0.217 (0.042-0.896) in the Asian population. Similarly, although there was no significant association between tea consumption and brain cancer risk, the pooled RR (95 % CI) of tea consumption for brain cancer risk was 0.798 (0.646-0.986) in the population of United States of America. Therefore, ethnic differences should be considered during the analysis. As glioma represents approximately a quarter of all brain cancers, risk stratification by the type of brain cancer is also required in a meta-analysis.

Regarding the mechanism of the association, the flavanols are the most important compound of tea polyphenols that predominantly include catechins, such as epicatechin, epicatechin gallate, epigallocatechin, epigallocatechin gallate, and catechin. Among them, Le et al. (2018[4]) reported that epigallocatechin-3-gallate might be a suitable adjuvant to potentiate anti-glioma therapies. In contrast, coffee has antioxidant characteristics, and coffee-related metabolites might be related to lowering incidence of glioma (Huang et al., 2017[3]). I suspect that the inconsistent epidemiological associations found between tea/coffee intake and glioma might be due to the complexity of lifestyle factors.

## Conflict of interest

The author declares no conflict of interest.
